# Unexpected Decrease in Milk Production after Fenbendazole Treatment of Dairy Cows during Early Grazing Season

**DOI:** 10.1371/journal.pone.0147835

**Published:** 2016-01-25

**Authors:** Nadine Ravinet, Christophe Chartier, Nathalie Bareille, Anne Lehebel, Adeline Ponnau, Nadine Brisseau, Alain Chauvin

**Affiliations:** 1 IDELE, French Livestock Institute, UMT Cattle Herd Health Management, Nantes, France; 2 LUNAM Université, Oniris, Nantes-Atlantic College of Veterinary Medicine and Food sciences and Engineering, UMR BioEpAR, Nantes, France; 3 INRA, UMR1300 Biology, Epidemiology and Risk Analysis in animal health, Nantes, France; Rega Institute for Medical Research, BELGIUM

## Abstract

Gastrointestinal nematodes (GIN) infection can impair milk production (MP) in dairy cows. To investigate whether MP would be optimized by spring targeted-selective anthelmintic treatment in grazing cows, we assessed (1) the effect on MP of an anthelmintic treatment applied 1.5 to 2 months after turn-out, and (2) herd and individual indicators associated with the post-treatment MP response. A randomized controlled clinical trial was conducted in 13 dairy farms (578 cows) in western France in spring 2012. In each herd, lactating cows of the treatment group received fenbendazole orally, control cows remained untreated. Daily cow MP was recorded from 2 weeks before until 15 weeks after treatment. Individual serum pepsinogen and anti-*Ostertagia* antibody levels (expressed as ODR), faecal egg count and bulk tank milk (BTM) *Ostertagia* ODR were measured at treatment time. Anthelmintic treatment applied during the previous housing period was recorded for each cow. In each herd, information regarding heifers’ grazing and anthelmintic treatment history was collected to assess the Time of Effective Contact (TEC, in months) with GIN infective larvae before the first calving. The effect of treatment on weekly MP averages and its relationships with herd and individual indicators were studied using linear mixed models with two nested random effects (cow within herd). Unexpectedly, spring treatment had a significant detrimental effect on MP (-0.92 kg/cow/day on average). This negative MP response was particularly marked in high producing cows, in cows not treated during the previous housing period or with high pepsinogen levels, and in cows from herds with a high TEC or a high BTM ODR. This post-treatment decrease in MP may be associated with immuno-inflammatory mechanisms. Until further studies can assess whether this unexpected result can be generalized, non-persistent treatment of immunized adult dairy cows against GIN should not be recommended in early grazing season.

## Introduction

Gastrointestinal nematode (GIN) infections are highly prevalent in young and adult grazing cattle [1–4]. The most frequent and pathogenic GIN in cattle is *Ostertagia ostertagi*, an abomasal worm responsible for clinical disease and growth retardation in young stock [5–9], and subclinical decrease in milk production (MP) in adult dairy cows [10–12].

Control measures are essential and depend heavily on blanket anthelmintic treatments, applied to whole groups or herds, to remove or prevent infection with GIN. Nevertheless, the regular use of anthelmintics exerts a heavy selection pressure on parasite populations leading to possible emergence of anthelmintic resistance. This risk is no longer negligible in cattle [13–19], threatening the long term efficacy of usual control programs aiming at securing growth of young stocks and MP in adult dairy cows. Changes in treatment practices are therefore needed, towards a more rational and sustainable use of anthelmintics.

In adult grazing dairy cows, looking at the large variability of the effect of anthelmintic treatment on MP among studies, between herds and between cows [10,11,20–26], there appears both a need and an opportunity to use anthelmintics in a more targeted and selective way. In targeted treatment (TT) and targeted-selective treatment (TST) strategies, treatments are restricted to herds, and cows within herds, that will most benefit from treatment (TST), drug being administered at the most appropriate time(s) (TT), bearing in mind the need to maintain susceptible parasites in *refugia* (parasites unexposed to drug, i.e. free-living stages on pasture, and parasitic stages within untreated hosts) [27–30]. The implementation of such treatment strategies against GIN should enable: (i) MP optimization, while (ii) limiting the use of anthelmintics, and (iii) lowering the risk of development of anthelmintic resistance thanks to the preservation of a susceptible parasite population in *refugia*.

TST administered during the winter housing period allows the preservation of a *refugia* population made up mainly of parasites within untreated hosts. Indeed, housed cows are no longer in contact with infective stages at that time, and the population of free-living stages on pasture decline in a substantial level during winter in cold temperate climate areas [5, 31]. Conversely, with TST during grazing season, the size of the *refugia* population could be theoretically greater, because it could be increased by the free-living stages on pasture resulting from spring parasitic cycles. To optimize MP in adult dairy cows while maintaining a large reservoir of susceptibility to anthelmintics in the GIN population, TST during grazing season, if it is associated with a significant increase in MP, could thus be optimal.

The effect of an anthelmintic treatment applied in autumn-winter (housing period) on MP has been broadly studied: it differs from one study to another, but even if it is sometimes non-significant or slight, a post-treatment increase in MP has often been observed [20–22, 25, 32–37]. In contrast, the MP response of anthelmintic treatment applied during the grazing season is less documented. Only one study reported a positive effect of a single treatment applied 1.5 months after turn out [38], but this study was conducted with a small sample size (40 cows in one herd). In a few other studies, several treatments were applied repeatedly on lactating cows during the grazing season, with a positive or a non-significant effect on MP [23, 39, 40]. But this whole herd repeated-treatment strategy is of course questionable if we keep in mind the need to preserve a large population of parasites in *refugia*. In other studies, a single treatment was applied, not in a given season and to the entire herd at the same time, but all over the year, at a given physiological stage, namely at calving or drying off [24, 26, 41–47]. Unfortunately, the majority of these studies did not provide a comparison of the effects on MP of treatments applied during housing period (autumn-winter) *versus* grazing season (spring, summer, and early autumn). Indeed, either the effect of season on the MP response after anthelmintic treatment was not investigated [24, 43, 45–47], or failed to be detected because of a possible lack of statistical power, as pointed out by the authors [44], or because seasons were not sufficiently distinguishable to be compared [41, 42]. Only one recent study reported that the treatment effects were similar during housing *versus* pasture period [26].

Consequently, the MP response after a single anthelmintic treatment during grazing season would deserve more in-depth examination, to determine whether such treatment strategies could offer a good compromise between the optimization of MP and the preservation of a large population of parasites in *refugia*.

The objectives of this study were (i) to assess the effect on MP of a single anthelmintic treatment applied at early grazing season, 1.5 to 2 months after turn out, (ii) to evaluate whether TST treatment could be possible at that time by analyzing the relationships between treatment response and several parasitological or production-based indicators, at both herd- and individual-levels.

## Materials and Methods

### Farms and animals

Thirteen dairy herds in the North-West of France were visited twice during spring 2012. The first visit took place around the date of turn out, and the second 1.5 to 2 months after turn out. Eleven of these herds were already included in a previous randomized controlled clinical field trial conducted in autumn 2011 [25]. The major herd recruitment criteria were the breed (Holstein), an access to pasture (with at least 1/3 of grazed grass in the cows’ diet), no intention of anthelmintic treatment on adult dairy cows in the upcoming grazing season, and a daily recording of MP (automatic milking system, or milk parlor with milk meters). In each herd, the majority of lactating cows were included provided that they were planned to be milked for at least 5 weeks after the second visit and in apparent good health.

This study was carried out in commercial dairy farms, so on "private lands". On the basis of an accurate description of the objectives of the study and its design, the owners of the lands (the farmers) gave permission to conduct the study on their farm, accepted to follow the protocol and to participate in the handling of cows. Except this informed consent of each farmer and his permission to conduct the study on his farm and on his animals, no other specific permission was required.

This field study did not involve endangered or protected species. No cows were sacrificed for the purposes of the study.

### Anthelmintic treatment

On the first visit, the history of anthelmintic treatment applied during autumn-winter 2011–2012 was recorded for each cow. In fact, some cows had actually been treated during the previous housing period either by the farmer, or by researchers in the context of the autumn clinical trial [25]. On the second visit, in each herd, cows were stratified according to three criteria: treated during the previous housing period (yes *versus* no), parity (first, second, third and greater), and days in milk (DIM) classes (less than 35 DIM, 35 to 100 DIM, 100 to 200 DIM, and more than 200 DIM). Then, in each stratum, cows were ranked and paired by ascending expected production level (last test-day milk yield before the second visit). Finally, cows of each pair were randomly assigned to a treatment group or a control group using a random number table. Cows belonging to the treatment group received orally on the day of the second visit a single 60 mL dose of fenbendazole (Panacur^®^ 10%), which is the dose for 800 Kg body weight (7.5 mg/Kg). Cows from the control group remained untreated.

### Samples and laboratory analysis

On the first visit, individual blood samples were taken from all cows (5mL blood sample at the coccygeal vein). On the day of treatment (second visit), individual blood and faecal samples (around 100g taken directly into the rectum, using gloves and lubricating gel) were taken from all treated and control cows, and a bulk tank milk (BTM) sample was collected in each herd.

Anti-*Ostertagia* antibody levels were determined with the ELISA SVANOVIR kit (Svanova Biotech, Uppsala, Sweden) on the individual blood samples and on the BTM samples. Results were expressed as an optical density ratio (ODR) [25]. Individual serum pepsinogen levels were also determined on blood samples, following the simplified method described by Kerboeuf et al. (2002) [48]. Results were expressed in milli-Units of Tyrosine (mUTyr). Individual faecal egg counts (FEC) per 5g of feces were determined using the modified Wisconsin Sugar Centrifugal flotation method [49].

This study was conducted by a veterinarian researcher who holds a licence to experiment on live animals (diploma issued by the National Veterinary School of Nantes (Oniris) which relies on the Ministère de l’Agriculture, de l’Agroalimentaire et de la Forêt). The protocol was painless for cows, all efforts were made to minimize stress and holding time, and every samples and handling were performed by a veterinarian, helped by the farmer when needed.

### Daily milk production data

Daily individual cow MP data were recorded and extracted from the farm computer from 14 days before treatment until 105 days after treatment. The average daily MP over the period of 14 days before treatment was calculated (one reference point for the pre-treatment period). Then, for the post-treatment period (15 weeks), daily MPs were averaged by week.

### Indicators characterizing cows and herds

Each cow was characterized by its daily MP averaged by week, and by 3 production-based indicators: parity, days in milk at the time of treatment (DIMt) and pre-treatment production level. To estimate this latter indicator, additional daily MP data, up to 49 days before the day of treatment, were recovered. The pre-treatment production level was then evaluated for each cow calculating the average daily MP over this period (maximum 49 days, and minimum 3 days according to DIMt).

Each cow was also characterized by 6 individual parasitological indicators: history of treatment applied during the previous housing period, serum pepsinogen levels and individual serum anti-*Ostertagia* antibody levels at the time of the first visit and on the day of treatment (second visit), and FEC on the day of treatment.

Each herd was characterized by its BTM anti-*Ostertagia* antibody level measured on the day of treatment. Moreover, on the basis of information regarding heifers’ grazing and anthelmintic treatment history, the Time of Effective Contact (TEC, in months) with GIN infective larvae before the first calving was calculated in each herd according to Ravinet et al. (2014) [25]: each herd was characterized by a minimal TEC (TECmin) and a maximal TEC (TECmax) according to its pattern of dates of birth and age at first calving.

### Statistical analysis

#### Coding and classification of variables

Raw data were entered into an Access database (Microsoft Corp., Redmond, WA). They were then transferred into SAS 9.2 (SAS Institute Inc., Cary, NC) to build the variables of interest and carry out statistical analyses.

The treatment was coded as described by Ravinet et al. (2014) [25]: a combined variable between treatment and time was created (‘week-trt’) and divided into 17 categories: for a treated cow, ‘week-trt’ took the values -1 (reference point before treatment), 0, 1, 2, 3 … up to 14 (after treatment period, week 0 being the week of treatment); and for a control cow, ‘week-trt’ only took the value 99 whatever the week.

The 3 production-based indicators were categorized. Three classes were constructed for parity (1, 2, 3 and more), and days in milk at the time of treatment (DIMt≤100, 100<DIMt≤200, DIMt>200 days). The pre-treatment production level was corrected for parity and days in milk and categorized in 3 classes (low, moderate, high) according to terciles of the distribution of the average MP calculated on the 3 to 49 days period before treatment (see above).

The 6 individual parasitological indicators were also categorized. Two classes were defined for FEC at the time of treatment (positive *versus* negative) and for the history of treatment applied during the previous housing period (yes *versus* no, whatever the drug used). For individual serum pepsinogen and anti-*Ostertagia* antibody levels measured on the first and on the second visit, three classes were defined according the terciles of their respective distribution.

At the herd-level, the BTM anti-*Ostertagia* antibody level was categorized in two classes, according to the median of its distribution. Two classes were also defined for TEC: high-TEC when TECmin ≥ 8 months (every heifers in the herd experienced a long TEC with GIN infective larvae before the first calving), and low-TEC otherwise (TECmax < 8 months, or TECmax ≥ 8 months but TECmin < 8 months).

To ensure that the number of treated cows and control cows was evenly distributed among the different categories of each indicator, the proportion of treated cows and control cows was compared using Chi-square tests (level of significance set at *p≤0*.*05*).

#### Comparison of individual parasitological indicators between cows treated or not treated during the previous housing period

Cows treated and not treated during the previous housing period were first compared on the basis of their individual serum pepsinogen levels and anti-*Ostertagia* antibody levels measured on the first visit around the date of turn out (parametric or non-parametric mean comparison tests according to the normality of the distribution of quantitative variables, level of significance set at *p≤ 0*.*05*).

These comparisons were also performed for the 3 parasitological indicators measured on the second visit (1.5 to 2 months after turn out) (chi-square tests for categorized variables, and parametric or non-parametric mean comparison tests according to the normality of the distribution of quantitative variables, level of significance set at *p≤0*.*05*).

#### Assessment of the overall treatment effect over time on milk production

The overall effect, over time, of spring treatment on daily MP averaged by week was studied using a first linear mixed model (model 0) with two nested random effects (cow within herd). The outcome variable was the daily MP averaged by week, expressed in Kg per day, and normally distributed (8393 observations, mean = 28.6 Kg/day, sd = 8.2 Kg/day). This model 0 included the following independent variables: week-trt (variable of interest, reference = 99), parity (1, 2, 3 or greater), days in milk (DIM) (expressed as week in milk: 1 to 52, and 52 or greater), pre-treatment production level corrected for parity and DIM (low, moderate, high), month of milk production (April to September), and a two way interaction between parity and DIM.

This model 0 was of the following form:

$$\begin{array}{ll} {\text{(Daily MP average by week)}}_{wij} & \text{= }\mu +\sum \beta_{wij}X_{wij}\text{+ }\sum \beta_{ij}X_{ij}\text{ + }\nu_{j}\text{ + }\omega_{ij}\text{ + }\varepsilon_{wij}\\ \text{with} & \\ & \nu_{j}∼N(0,\sigma_{\nu }^{2}),\;\omega_{ij}∼N(0,\sigma_{\omega }^{2})\\ & \varepsilon_{wij}=\left[\varepsilon_{1}{}_{ij},\varepsilon_{2ij},...,\varepsilon_{14ij}\right]∼N(0,\sigma_{\varepsilon }^{2}) \end{array}$$

where (Daily MP averge by week)_*wij*_ = MP for cow *i* in farm *j*, on week *w*, *μ* = average MP after adjusting for covariates, *β*_*wij*_ = coefficients for *X*_*wij*_, *X*_*wij*_ = variables varying between daily MP averaged by week (DIM, week-trt, month of milk production, and DIM*parity), *β*_*ij*_ = coefficients for *X*_*ij*_, *X*_*ij*_ = variables varying between cows (parity, pre-treatment production level), *v*_*j*_ = farm random effect, *ω*_*ij*_ = cow random effect nested into herd, *ε*_*wij*_ = residual at week *w*. The random effects *v*_*j*_, *ω*_*ij*_ and the residual *ε*_*wij*_ were assumed to be normally distributed with mean 0 and variance $\sigma_{\nu }^{2}$, $\sigma_{\omega }^{2}$ and $\sigma_{\varepsilon }^{2}$ respectively.

Residuals and predicted values were plotted to evaluate their heteroscedasticity and their normality.

#### Assessment of the variations of the treatment effect according to indicators characterizing herds and cows on the second visit

The relationships between the treatment response and (i) the 3 categorical individual production-based indicators, (ii) the 3 categorical individual parasitological indicators measured on the second visit, plus the history of treatment applied during the previous housing period, and (iii) the 2 categorical herd-level indicators were evaluated using one linear mixed model per indicator. Each of these models included the same independent variables as the model 0, plus the indicator of interest in interaction with week-trt. For each of these models, residuals and predicted values were plotted to evaluate their heteroscedasticity and their normality.

#### Estimation method to assess the milk production gain week after week

The statistical models estimated each week the average daily MP of treated cows *versus* control cows, for the whole population of our study sample (model 0), or within each category of the investigated indicators (models including the interaction terms between week-trt and indicators). On the basis of these estimations, the treated cows’ MP gain was calculated each week as described in Ravinet et al. (2014) [25]. Finally, with G_i_ being the estimated treated cows’ MP gain in week_i_, the average treated cows’ MP gain for the whole period of follow up after treatment (from week_0_ to week_14_) was the arithmetic mean of the G_i_s.

To test each week if G_i_s were significantly different from zero, we used student tests with adjusted p-values for multiple testing.

## Results

### Description of the study sample

625 cows in 13 herds were initially included in the study. 11 herds were equipped with an automatic milking system, and 2 with a milking parlor with milk meters (herd n°1 and 9). General information regarding spring grazing practices of dairy cows in our study sample is provided in Table 1. Cows were turned out between 2012-02-28 and 2012-03-28. The proportion of grazed grass in the cows’ spring diet increased gradually from turn out onwards (from 20% to 75%) (Table 1), and was not lower in herds with automatic milking systems. The first visits took place between 2012-03-07 and 2012-03-28. In five herds it was on the day of turn out, or 2 to 13 days before, whereas in 7 herds it was 6 to 14 days after. The time lag between turn out and this first visit was longer (25 days) only for one herd (herd n°13) (Table 1). Anthelmintic treatment was administered between 2012-04-16 and 2012-05-29, that is to say 41 to 68 days after turn out. In two herds (n°4 and 10), the actual time spent on pasture between turn out and treatment was shorter (37 and 34 days, respectively) because cows returned to the barn for 4 and 1 weeks respectively, because of adverse weather conditions.

**Table 1:** Dates of visits and description of the spring grazing management practices of dairy cows in the 13 herds included in the study carried out on spring 2012.

N° herd	Date of turn out	Date of first visit	Time lag between turn out and first visit (days)	Date of treatment (second visit)	Time lag between turn out and treatment (days)	% of grazed grass in the spring cows’ diet	Grazing time per day (hours)*	Number of plots grazed by dairy cows in spring (size)	Time spent on each plot (days)	Average number of lactating cows
1	03–28	03–28	0	05–16	49	33 to 66%	6	4 (1.5 to 4 ha)	8	65
2	03–23	03–19	-4	05–24	62	50 to 66%	19*	3 (4.5 ha)	5 to 6	60
3	03–02	03–16	14	05–03	62	50 to 70%	4 à 6	7 (3 to 4 ha)	5	70
4	03–26	03–13	-13	05–29	64^1^	20 to 50%	13*	2 (4 ha)	2	40
5	03–15	03–13	-2	05–22	68	30 to 40%	24*	5 (1.2 ha)	5	50
6	03–01	03–07	6	04–24	54	30 to 60%	10*	3 (8 to 10 ha)	15	70
7	03–13	03–23	10	05–09	57	30 to 75%	24*	9 (0.5 to 2 ha)	2 to 7	47
8	03–02	03–08	6	04–26	55	30%	9*	5 (2 ha)	3	47
9	03–12	03–22	10	05–14	63	30%	4	3 (2.5 to 4 ha)	1	120
10	03–22	03–12	-10	05–02	41^2^	30 to 50%	12*	5 (0.7 to 1.6 ha)	3 to 4	40
11	03–14	03–21	7	05–07	54	30 to 50%	17*	6 (1 to 3 ha)	6 to 10	50
12	03–01	03–14	13	04–18	48	15 to 65%	NA^3^	14 (1 ha)	2 to 4	70
13	02–28	03–24	25	04–16	48	25 to 70%	9 à 24*	5 (2 to 3 ha)	7 to 15	50

*In herds where the barn is always open (access to the automatic milking system, or access to feed in the trough), cows freely move from the barn to the pasture. The information given here is thus the duration of free access to pasture per day

^1^In this herd, cows returned to the barn between 04–18 and 05–15, the actual time spent on pasture between turn out and treatment was thus 37 days.

^2^In this herd, cows returned to the barn between 04–25 and 05–02, the actual time spent on pasture between turn out and treatment was thus 34 days.

^3^NA ^=^ Not available.

47 cows were excluded from the dataset for the following reasons: insufficient number of MP data recovered before treatment to calculate the pre-treatment production level (10 cows), days in milk at the time of treatment exceeding 516 days (11 cows), daily MP data not correctly recorded on farm (1 cow), duration of the post-treatment follow-up shorter than 4 weeks or health issue indicated by the farmer (25 cows).

Finally, 578 cows were included to carry out our statistical analysis: 295 treated cows and 283 control cows, 31 to 72 cows per herd. The final dataset is described in Tables 2–4. Among these 578 cows, 556 were present on the two visits, and 22 were sampled only on the second visit. Thus, serum pepsinogen levels and anti-*Ostertagia* antibody levels measured on the first visit were not available for these 22 cows.

**Table 2:** Description of the dataset (578 cows in 13 herds, 295 treated cows, 283 control cows): distribution and classification of individual production-based indicators characterizing cows, number of treated cows and control cows per classes for each categorized indicator, and distribution of the daily milk production average by week.

							Classes threshold and number of cows per classes				
Indicator	Minimum	Q1	Median	Q3	Maximum	Mean *(std)*		Control cows	Treated cows	Total	*Chi-square test*
Parity	1	1	2	3	8	2.1 *(1*.*2)*	1	111	110	221	*p = 0*.*89*
							2	94	102	196	
							3 and greater	78	83	161	
Days in milk at the time	4	89	193	260	516	187 *(114)*	DIMt ≤ 100	79	83	162	*p = 0*.*91*
of treatment (days)							100 < DIMt ≤ 200	68	75	143	
(DIMt)							DIMt > 200	136	137	273	
Production level* (kg/day)	13.5	26.8	32.3	38.6	62.4	32.8 *(8*.*1)*	Low	87	92	179	*p = 0*.*98*
							Moderate	94	96	190	
							High	102	107	209	
Daily milk production average by week	5.2	22.8	27.9	33.9	61.6	28.6 *(8*.*2)*	-	-	-	-	-

*Average daily MP calculated on the 3 to 49 days period before treatment and corrected for parity and days in milk.

**Table 3:** Description of the dataset (578 cows in 13 herds, 295 treated cows, 283 control cows): distribution and classification of individual parasitological indicators characterizing cows, number of treated cows and control cows per classes for each categorized indicator.

							Classes threshold and number of cows per classes				
Indicator	Minimum	Q1	Median	Q3	Maximum	Mean *(std)*		Control cows	Treated cows	Total	*Chi-square test*
Serum pepsinogen level	243	1270	1622	2042	4391	1677 *(592)*	Pepsi_1_ ≤ 1401^2^	88	100	188	*p = 0*.*31*
on the first visit^1^							1401< Pepsi_1_ ≤ 1815	95	82	177	
(Pepsi_1_)(mUTyr)							Pepsi_1_ > 1815^2^	89	102	191	
Serum pepsinogen level	350	1258	1762	2265	5509	1878 *(847)*	Pepsi_2_ ≤ 1419^2^	93	90	183	*p = 0*.*61*
on the second visit^3^							1419 < Pepsi_2_ ≤ 2025	99	98	197	
(Pepsi_2_) (mUTyr)							Pepsi_2_ > 2025^2^	91	106	197	
Anti-*Ostertagia* antibody	-0.10	0.37	0.56	0.73	1.26	0.54 *(0*.*27)*	ODR_1_ ≤ 0.452^2^	87	108	195	*p = 0*.*32*
level on the first visit^1^							0.452 < ODR_1_ ≤0.670	89	87	176	
(ODR_1_)							ODR_1_ > 0.670^2^	96	89	185	
Anti-*Ostertagia* antibody	-0.081	0.22	0.48	0.64	1.10	0.43 *(0*.*28)*	ODR_2_ ≤ 0.309^2^	89	93	182	*p = 0*.*45*
level on the second visit^3^							0.309 < ODR_2_ ≤ 0.578	89	105	194	
(ODR_2_)							ODR_2_ > 0.578^2^	105	96	201	
FEC on the second visit	0	0	0	1	66	1.5 *(4*.*8)*	Positive FEC	99	98	197	*p = 0*.*55*
(eggs per 5g of feces)^4^							Negative FEC	179	197	376	
Treatment during the	-	-	-	-	-	-	Yes	106	122	228	*p = 0*.*34*
previous housing period							No	177	173	350	

^1^ 22 missing data

^2^ Terciles of the global distribution of the quantitative variable

^3^ 1 missing data

^4^ 5 missing data.

**Table 4:** Description of the dataset (578 cows in 13 herds, 295 treated cows, 283 control cows): distribution and classification of indicators characterizing herds, number of treated cows and control cows per classes for each categorized indicator.

							Classes threshold and number of cows (and herds) per classes				*Chi-square*
Indicators	Minimum	Q1	Median	Q3	Maximum	Mean *(std)*		Control cows	Treated cows	Total	*test*
TEC^5^ with GIN larvae before first calving	3	6.5	8.5	10.4	15	8.5 (*3*)	High-TEC (TEC_min_ ≥ 8)	118	127	245 (5 herds)	*p = 0*.*74*
(months)							Low-TEC (otherwise)	165	168	333 (8 herds)	
Bulk tank milk anti-*Ostertagia* antibody level	0.546	0.652	0.717	0.863	0.994	0.766 *(0*.*151)*	BTM ODR < 0.717^6^	152	150	302 (7 herds)	*p = 0*.*72*
on the second visit (BTM ODR)							BTM ODR ≥ 0.717	133	143	276 (6 herds)	

^5^TEC = Time of Effective Contact

^6^Median of the between-herd distribution.

Several cows were dried off or sold during the period of post-treatment follow-up. The number of cows therefore decreased gradually from one week to another, similarly in treated cows and control cows. On week_14_, there were still 428 cows in the dataset (226 treated cows and 202 control cows).

For each indicator, there was no difference in the proportion of treated cows between categories (*p>0*.*05*) (Tables 2–4).

### Comparison of individual parasitological indicators between cows treated or not treated during the previous housing period

#### At the time of the first visit

On the first visit, on average, serum pepsinogen levels tended to be slightly higher in cows not treated during the previous housing period compared to cows treated during the previous housing period (1708 *versus* 1632 mUTyr, *p = 0*.*07*). In the five herds sampled before or on the day of turn-out, this difference was not significant (1575 *versus* 1600 mUtyr, *p = 0*.*7*). In contrast, in the 8 herds sampled 6 to 25 days after turn-out, this difference was significant: cows not treated during the previous housing period had a higher serum pepsinogen level than cows treated during this autumn-winter period (1770 *versus* 1645 mUTyr, respectively, *p = 0*.*02*).

On average, anti-*Ostertagia* antibody levels were not different between cows treated or not during the previous housing period (average ODR = 0.53 and 0.54 respectively, *p = 0*.*32*), whatever the date of first visit compared to the date of turn-out.

#### At the time of the second visit

Table 5 describes the individual parasitological indicators measured 1.5 to 2 months after turn-out according to the history of anthelmintic treatment applied during the previous housing period. Anti-*Ostertagia* antibody levels and pepsinogen levels were on average significantly higher in cows not treated *versus* cows treated during the previous housing period (*p < 0*.*0001*). Moreover, those previously untreated cows had more often a positive FEC (39% *versus* 28%, *p = 0*.*007*).

**Table 5:** Comparison of individual parasitological indicators measured 1.5 to 2 months after turn-out (second visit, spring 2012) according to the history of anthelmintic treatment applied during the previous housing period (autumn-winter 2011–2012).

Treatment during the previous housing period	NO	YES	*Mean comparison test*	*Chi-square test*
Anti-*Ostertagia* antibody level	*n = 350 cows*	*n = 227 cows*		
(ODR)	mean = 0.48 (*std = 0*.*26*)	mean = 0.36 (*std = 0*.*29*)	*p < 0*.*0001*	-
	Q1 = 0.30; Q2 = 0.51; Q3 = 0.66	Q1 = 0.12; Q2 = 0.40; Q3 = 0.59		
Serum pepsinogen level (mUtyr)	*n = 350 cows*	*n = 227 cows*		
	mean = 1997 mUtyr (*std = 858*)	mean = 1694 mUtyr (*std = 797*)	*p < 0*.*0001*	-
	Q1 = 1390; Q2 = 1855; Q3 = 2493 mUtyr	Q1 = 1107; Q2 = 1587; Q3 = 2025 mUtyr		
FEC (positive *versus* negative)	*n = 346 cows*	*n = 227 cows*		
	Positive: 134 *cows (39%)*	Positive: 63 *cows (28%)*	-	*p = 0*.*007*
	Negative: 212 *cows (61%)*	Negative: 164 *cows (72%)*		

### Overall milk production response after spring anthelmintic treatment over time

**Fig 1:**
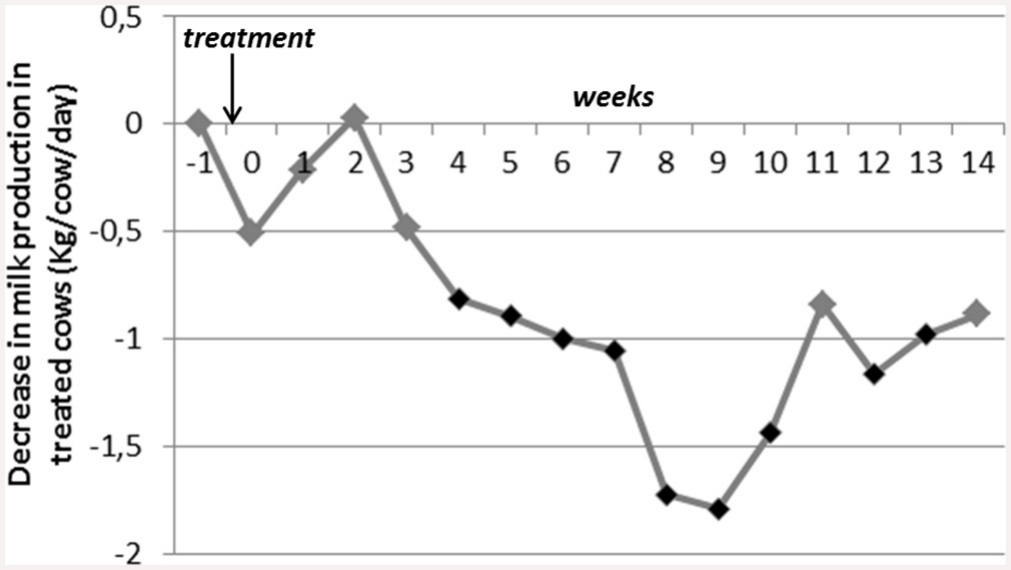
Evolution of the treated cows’ milk production over time in comparison with control cows (G_i_s < 0) (model 0, n = 578 cows, 295 treated cows, 283 control cows). Week_0_ = week of treatment, the first day of week_0_ is the day of treatment. Black dots on the curve indicate that the corresponding negative G_i_ are significantly different from zero, adjusted p-value<0.05: the drop in milk production for treated cows is significant during these weeks).

In model 0, all the usual factors associated with the variations of MP were significant (parity, DIM, interaction between parity and DIM, pre-treatment production level, and month of milk production) (*p<0*.*0001*). The combined variable of interest ‘week-trt’ was significant (*p < 0*.*0001*), and the effect of spring treatment on MP was negative: in comparison with the 283 control cows, the 295 treated cows’ daily MP averaged by week decreased significantly (G_i_s were negative). The evolution of the treated cows’ MP over time is displayed in Fig 1. The drop in MP after treatment was sharp: -0.92 Kg/cow/day on average for the whole period of follow up after treatment, with a maximum of -1.8 Kg/cow/day on week_9_ after treatment (Fig 1).

### Variations of the milk production response after spring anthelmintic treatment according to the indicators characterizing cows and herds

#### Variations of the treatment response according to individual production-based indicators

**Fig 2:**
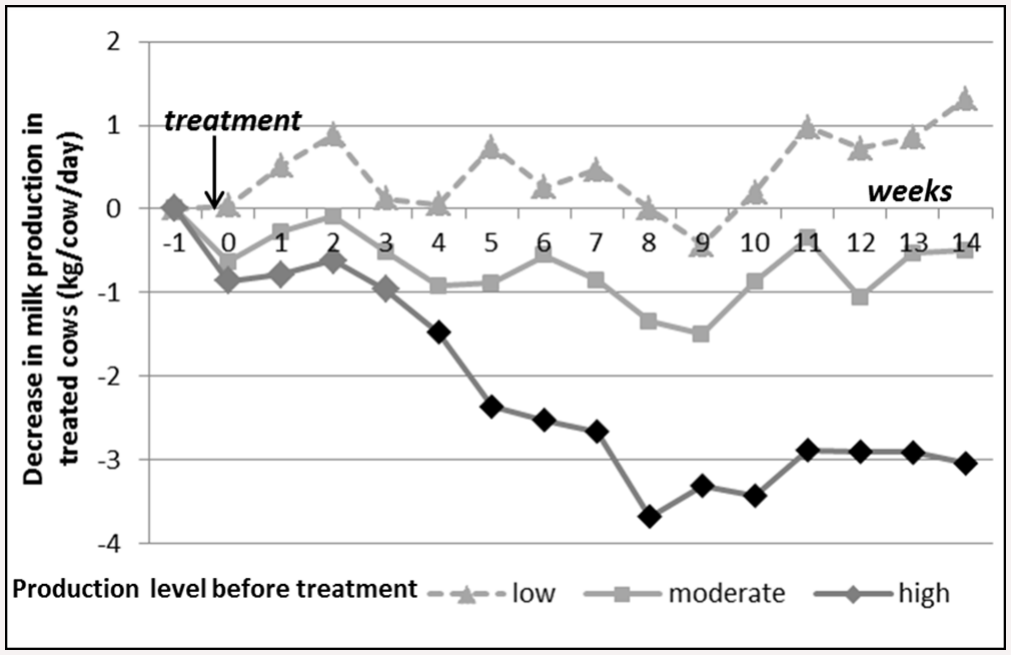
Evolution of the treated cows’ milk production over time in comparison with control cows (G_i_s) according to the pre-treatment production level (corrected for parity and days in milk). Week_0_ = week of treatment, the first day of week_0_ is the day of treatment. Black dots on the curve indicate that the corresponding negative G_i_ are significantly different from zero, adjusted p-value<0.05: the drop in milk production for treated cows is significant during these weeks). See Table 2 for the number of treated cows and control cows per category.

The interaction between week-trt and pre-treatment production level was significant (*p < 0*.*0001*). The high-producing treated cows’ daily MP averaged by week decreased significantly and markedly compared to the one of high-producing control cows (-2.3 Kg/cow/day on average, until -3.7 Kg/cow/day on week_8_ after treatment), whereas moderate and low-producing treated cows did not experience significant losses in milk production after treatment (Fig 2).

Interactions between parity or days in milk at the time of treatment and week-trt were not significant (*p = 0*.*44* and *p = 0*.*41*, respectively).

#### Variations of the treatment response according to individual parasitological indicators

**Fig 3:**
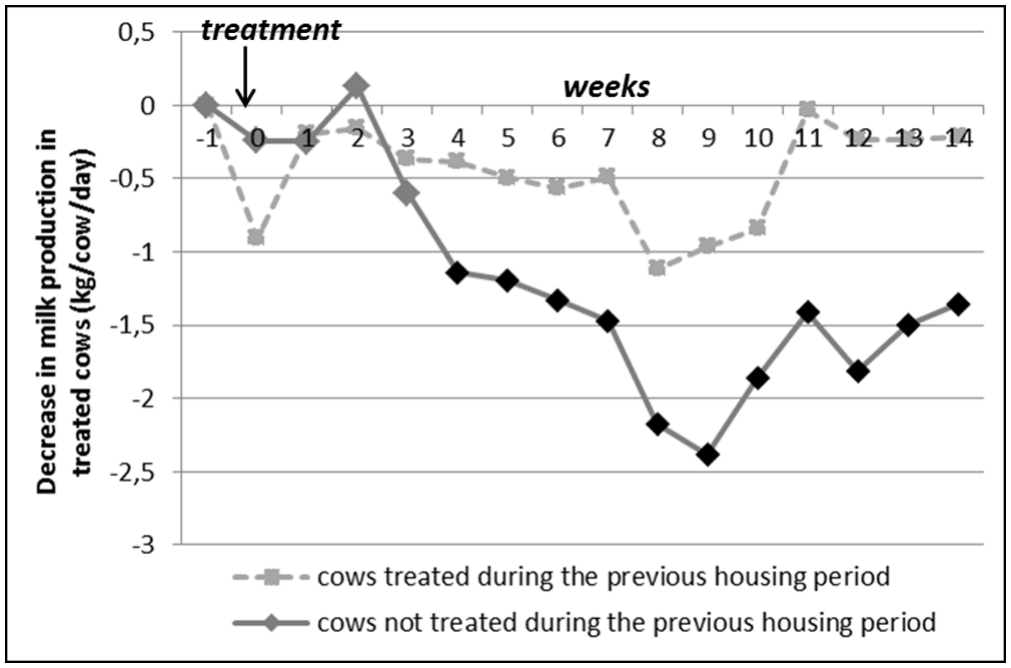
Evolution of the treated cows’ milk production over time in comparison with control cows (G_i_s) according to the history of anthelmintic treatment (treated *versus* not treated during the previous housing period). Week_0_ = week of treatment, the first day of week_0_ is the day of treatment. Black dots on the curve indicate that the corresponding negative G_i_ are significantly different from zero, adjusted p-value<0.05: the drop in milk production for treated cows is significant during these weeks).—See Table 3 for the number of treated cows and control cows per category.

The evolution of MP after spring treatment differed significantly between cows which had been treated during the previous housing period and cows which had not been treated during this autumn-winter period (interaction between week-trt and history of anthelmintic treatment: *p < 0*.*0001*). The decrease in MP after spring treatment was marked and significant in cows not previously treated during the housing period (-1.2 Kg/cow/day on average, until -2.4 Kg/cow/day on week_9_ after spring treatment), whereas this decrease remained non-significant in cows previously treated during the housing period (Fig 3).

**Fig 4:**
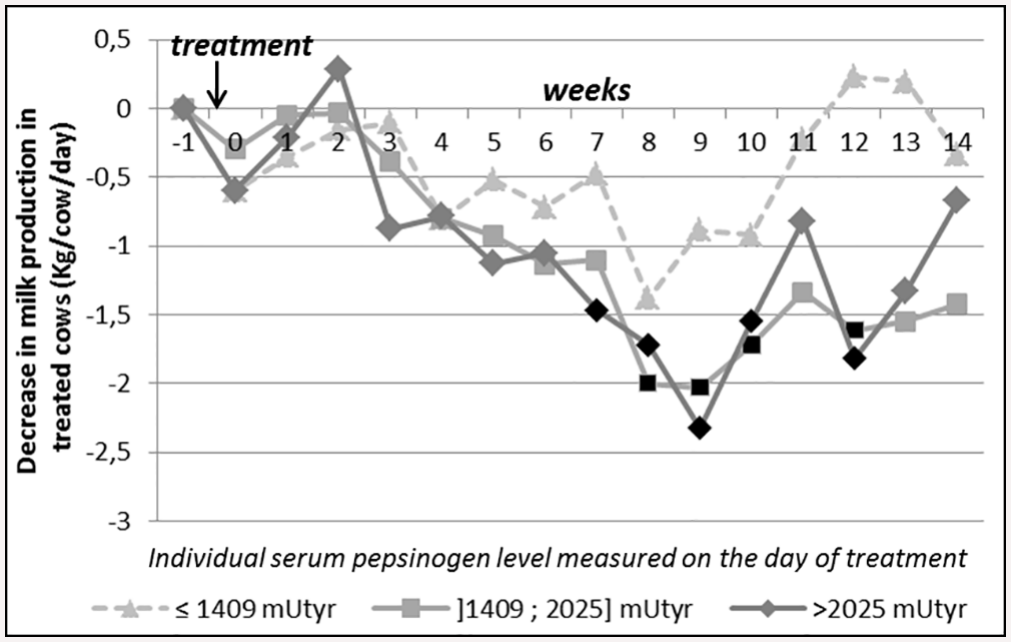
Evolution of the treated cows’ milk production over time in comparison with control cows (G_i_s) according to serum pepsinogen level at the time of treatment (second visit, 1.5 to 2 months after turn-out). Week_0_ = week of treatment, the first day of week_0_ is the day of treatment. Black dots on the curve indicate that the corresponding negative G_i_ are significantly different from zero, adjusted p-value<0.05: the drop in milk production for treated cows is significant during these weeks). See Table 3 for the number of treated cows and control cows per category.

Serum pepsinogen level measured on the second visit was significantly associated with the evolution of MP after treatment (interaction between week-trt and pepsinogen level 1.5 to 2 months after turn-out: *p < 0*.*0001*). Moderate and high-pepsinogen level cows (between 1409 and 2025 mUTyr, and > 2025 mUTyr, respectively) experienced higher MP losses after treatment than low-pepsinogen level cows: -1.1 Kg/cow/day on average for moderate and high-pepsinogen level cows, whereas the post-treatment decrease in MP was not significant in low-pepsinogen level cows (Fig 4).

FEC and anti-*Ostertagia* antibody level measured at the time of treatment were not associated with the evolution of MP after treatment: interactions between these two variables and the combined variable week-trt were not significant (*p = 0*.*21* and *p = 0*.*81*, respectively).

#### Variations of the treatment response according to herd-level indicators

**Fig 5:**
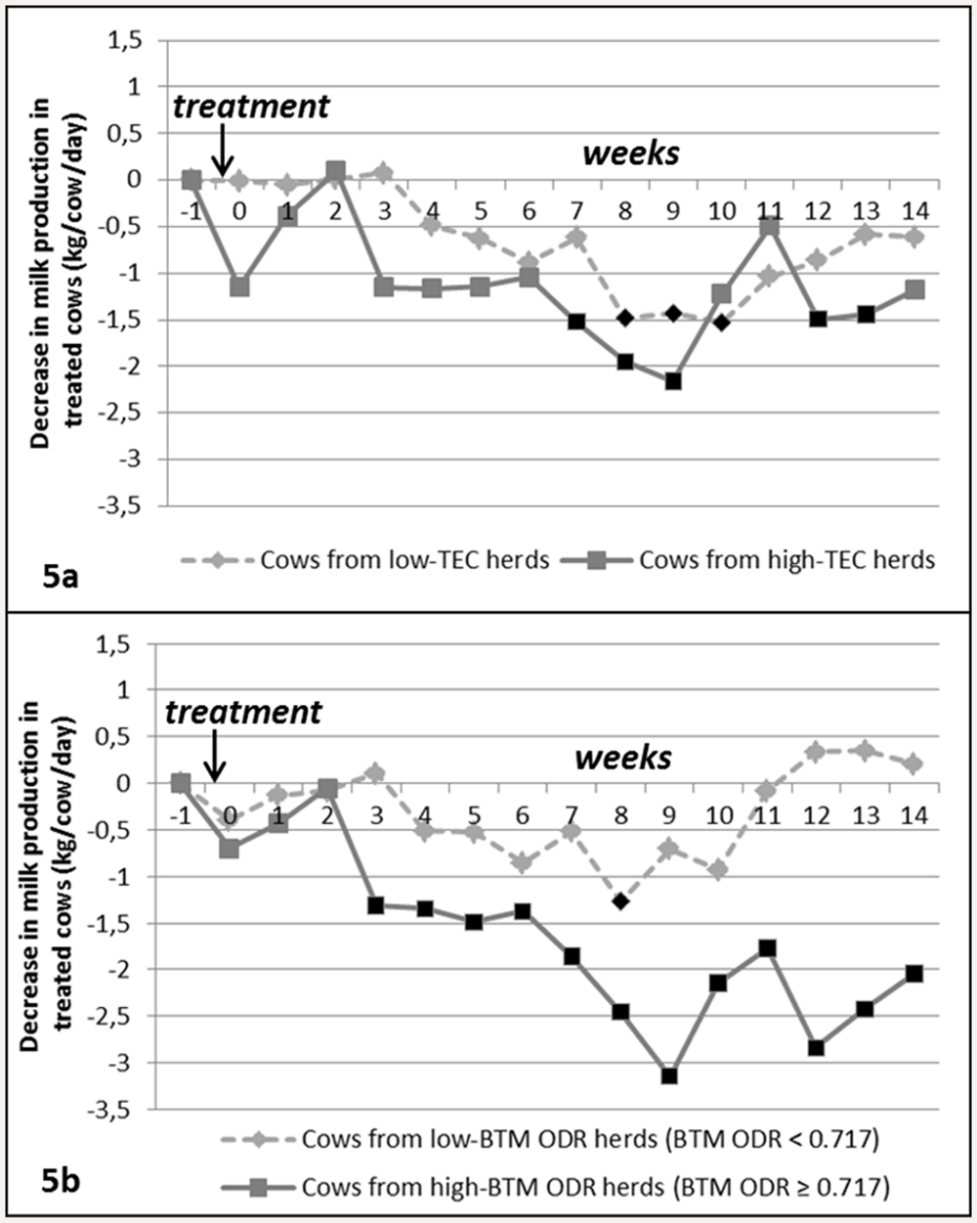
Evolution of the treated cows’ milk production over time in comparison with control cows (G_i_s) according to the 2 herd-level indicators (n = 578 cows): 5a) Time of Effective Contact (TEC) with GIN infective larvae before the first calving, 5b) Anti-*Ostertagia* antibody level in the bulk tank milk (BTM ODR). Week_0_ = week of treatment, the first day of week_0_ is the day of treatment. Black dots on the curve indicate that the corresponding negative G_i_ are significantly different from zero, adjusted p-value<0.05: the drop in milk production for treated cows is significant during these weeks). See Table 4 for the number of treated cows and control cows per category.

Fig 5 displays the evolution of the treated cows’ MP in comparison with control cows’ MP (G_i_s) according to the two herd-level indicators. The interaction between week-trt and TEC was significant (*p = 0*.*001*): after treatment, the MP of cows from high-TEC herds decreased somewhat more markedly than the MP of cows from low-TEC herds (-1.2 *versus* -0.7 Kg/cow/day, on average) (Fig 5a). The interaction between week-trt and BTM ODR was also significant (*p < 0*.*0001*): cows from high-BTM ODR herds (BTM ODR ≥ 0.717) experienced significant MP losses after treatment (-1.7 Kg/cow/day on average, down to -3.1 Kg/cow/day on week_9_ after treatment), whereas the decrease in MP for treated cows from low-BTM ODR herds (BTM ODR < 0.717) remained much lower (-0.33 Kg/cow/day, down to -1.3 Kg/cow/day on week_8_ after treatment) (Fig 5b).

## Discussion

This is the first study dealing with the effect on milk production (MP) of a single non-persistent anthelmintic treatment (fenbendazole) applied on lactating cows in spring, in the first part of the grazing season, and taking into account the history of treatment administered during the previous housing period. By treating 1.5 to 2 months after turn-out, one could expect (i) either a positive effect on MP due the removal of parasites from hosts, with in addition the preservation of a parasite population in *refugia* (free-living stages on pastures resulting from the spring development of eggs excreted since turn-out), (ii) or an absence of effect on MP due the possible rapid re-infection after this non-persistent anthelmintic treatment (persistent exposure to parasites as cows are grazing). Therefore, this prolonged negative marked effect of spring treatment on MP is quite surprising and contrary to what was initially expected. This negative effect made it impossible to identify the categories of cows that could be treated selectively in spring (TST strategy) on the basis of the demonstration of their improved milk production after treatment. However, a possible explanation of this negative effect of the treatment may be found by looking at the factors associated with the treatment response. The decrease in MP was particularly marked in cows (i) with a high pre-treatment production level, (ii) not treated with anthelmintics during the previous housing period (autumn-winter 2011–2012), (iii) with moderate or high pepsinogen level at the time of spring treatment (pepsinogen level being associated with the history of anthelmintic treatment), (iv) from herds with a high bulk tank milk anti-*Ostertagia* antibody level (BTM ODR > 0.717, median of our sample), and (v) from high-TEC herds.

Studies investigating the effect of a benzimidazole treatment on MP sometimes reported negative values, but the decrease in MP always remained non-significant [10, 11], except in one Norwegian study, conducted in 22 dairy herds, where fenbendazole treatment was applied at calving from September to the end of January, while cows were housed [50]. In this previous Norwegian study, the significant reduction in milk yield of the fenbendazole-treated cows was -151 kg in the subsequent lactation. The authors did not put forward any hypothesis to explain this decrease in MP. Jara et al. (1984) [51] showed that albendazole, fenbendazole and oxfendazole could cause a decrease in volatile fatty acid concentration and digestibility of cellulose in the rumen of sheep. However, according to Tharaldsen and Helle (1989) [50], those effects of fenbendazole treatment are unlikely to influence significantly MP for several consecutive weeks, in light of its rapid elimination from the body.

Forbes et al. (2004) [38] reported a significant positive MP response on weeks 2 and 3 after a single anthelmintic treatment applied, as in our study, 1.5 month after turn-out. However, in this previous study, cows were treated with eprinomectin, a persistent-activity product which prevents from re-infection with *Ostertagia* for 28 days [52]; and MP was followed for only 4 weeks after treatment, a period over which cows could therefore not be re-infected. The same was true in studies where several treatments were applied repeatedly on lactating cows during the grazing season [23, 39, 40]: positive effects of these repeated treatment strategies on MP were mainly reported but with highly limited post-treatment reinfections during the period of follow-up. On the contrary, in our study, treated cows could be re-infected during the 105 days period of follow up after the non-persistent fenbendazole treatment, because they kept grazing together with control cows on pastures where parasitic cycles had been re-initiated for 1.5 to 2 months. As a result, it may be assumed that the prolonged negative marked effect of spring treatment on MP was associated with post-treatment re-infection. This hypothesis is supported by the more severe and significant post-treatment decrease in MP observed in cows from high-BTM ODR herds at the time of treatment, i.e. from herds with a higher exposure to and therefore more prone to re-infection, due to a higher pasture infectivity [53].

Moreover, our observations suggest that immunological and inflammatory mechanisms may be associated with this negative effect of spring treatment on MP. Indeed, the negative post-treatment MP response was more marked in herds where the higher exposure to GIN could have induced a heavier antigenic stimulation (high BTM ODR herds). The negative post-treatment MP response was also somewhat more marked in cows from high-TEC herds, i.e. in herds where the acquired immune response controlling the establishment of ingested L3 could be stronger [25, 54], causing a stronger reaction when new contact with L3 occurred. This phenomenon is known in resistant sheep: a new larval challenge can induce a strong inflammatory reaction, with clinical signs of diarrhea, even though worm burdens are low [55–59]. As it leads to the elimination of worms, this phenomenon is commonly described as “self-cure”. It has been demonstrated that it is based on mechanisms similar to type I hypersensitivity [60], explaining the deleterious consequences for hosts. In cattle, high pepsinogen levels observed in second-grazing-season cattle exposed to an initial low level of infection [5, 31] and clinical forms of oedematous ostertagiosis [61] are also considered as deleterious effects of these particular immuno-inflammatory processes in immune animals.

This hypothetical association between inflammatory processes and negative MP response is also supported by the fact that the post-treatment decrease in MP was particularly marked in cows not treated during the previous housing period. In such cows, which contributed the most to the overall negative spring treatment response, the inflammatory state of the abomasal mucosa could have been more pronounced. Indeed, their worm burden is expectedly higher than the one of cows treated during the previous housing period, resulting in the resumption of development of a larger number of inhibited larvae, with higher damage of the abomasal mucosa (as evidenced by higher pepsinogen levels found in these cows). In addition, serum pepsinogen levels of these cows were higher when they were measured after turn-out, particularly on the second visit 1.5 to 2 months after turn-out, demonstrating higher abomasal mucosal damages potentially also caused by the response to larval challenge between turn-out and the date of anthelmintic treatment.

Those inflammatory processes may have been exacerbated by the persistence of dead *Ostertagia* larvae in the abomasal mucosa following fenbendazole treatment. Two studies come in support of this hypothesis. (i) In yearling beef cattle naturally infected with *O*. *ostertagi*, treated with fenbendazole while a large number of early fourth stage inhibited larvae had been established, and then slaughtered 7 to 12 days after treatment, Snider III et al. (1985) [62] observed an aggravation of the abomasal inflammatory lesions compared to those found in untreated calves. This aggravation was related to the persistence of dead and degenerate *O*. *ostertagi* larvae in the abomasal mucosa of fenbendazole treated calves. Indeed, these degenerate larvae were not observed in the tissues of untreated calves nor in calves treated with ivermectin, and were thus attributed to the fenbendazole treatment. Histologically, the presence of a marked eosinophil and lymphocyte infiltration and of an unidentified material coating the degenerate larvae suggested an inflammatory and/or immunological response initiated or exacerbated by the presence of such larvae [62]. (ii) Similar results were found in horses naturally infected with cyathostomins and treated either with fenbendazole or moxidectin [63]: in contrast to moxidectin effects, the killing of larvae due to fenbendazole treatment was associated with severe tissue damage. These observations argue in favor of the involvement of fenbendazole treatment in the maintenance of inflammatory reaction of the abomasal mucosa. However, considering the persistence of the negative effect of treatment on MP, one may wonder what the duration of the pro-inflammatory effect of dead larvae is. Snider III et al. (1985) [62] indicated that these degenerate *Ostertagia* larvae were found up to 20 days after treatment, but in smaller proportion than between 7 to 12 days after treatment, and that the time needed for dead nematode clearance is unknown for *O*. *ostertagi* and other GIN. Moreover, other observations are in favour of the pro-inflammatory effect of larvae killed by the treatment. In the study conducted by Ravinet et al. (2014) [25], where adult dairy cows were treated with fenbendazole at housing and where the pattern/kinetics of the treatment response is described, the global effect of treatment on MP was positive but was not immediate, and treated cows tended to produce less milk than control cows during the first two weeks following the date of treatment. This could suggest a slight deleterious effect of treatment, before tissue repair and increased feed intake following the removal of parasites could allow a moderate increase in MP [25].

Further studies are needed to assess whether this unexpected negative effect of spring anthelmintic treatment on MP can be generalized, and to identify and understand the biological mechanisms underlying this decrease in MP. A study investigating more closely the inflammatory and immune responses would be necessary to assess the potential adverse effects of (i) the larval challenge at turn-out, and of (ii) the spring anthelmintic treatment under different exposures to GIN, and with different immunological status (TEC). Moreover, the approach outlined in this study should be replicated with another anthelmintic (eprinomectin), to assess whether our observed negative effect of treatment is related, at least in part, to the use of fenbendazole. Finally, a longer post-treatment follow-up of MP would enable to assess if this deleterious effect of treatment on MP is reversible and how long it lasts.

## Conclusion

The idea of optimizing milk production while preserving a large GIN population in *refugia* by spring TST in grazing dairy cows ran up against an unexpected result: the anthelmintic treatment applied 1.5 to 2 months after turn-out had a significant negative effect on milk production.

Our result suggests that treatment for GIN of dairy cows cannot be generally recommended and must be associated with seasonal considerations. Until further studies can assess whether this unexpected result can be generalized or is tied to specific conditions, anthelmintic treatment of adult dairy cows immunized against GIN, specifically non-persistent treatment, should be discouraged at the beginning of the grazing season, and should rather be targeted in autumn at housing.
